# The Tumor Invasion Paradox in Cancer Stem Cell-Driven Solid Tumors

**DOI:** 10.1007/s11538-022-01086-4

**Published:** 2022-10-27

**Authors:** Alexandra Shyntar, Ashna Patel, Meghan Rhodes, Heiko Enderling, Thomas Hillen

**Affiliations:** 1 University of Alberta, Edmonton, Canada; 2 Moffitt Cancer Center, Tampa, USA

**Keywords:** Mathematical oncology, Cancer modeling, Cancer stem cells, Intermittent therapy, Tumor growth paradox, 92B05, 92C37, 92C50, 37N25

## Abstract

Cancer stem cells (CSCs) are key in understanding tumor growth and tumor progression. A counterintuitive effect of CSCs is the so-called *tumor growth paradox*: the effect where a tumor with a higher death rate may grow larger than a tumor with a lower death rate. Here we extend the modeling of the tumor growth paradox by including spatial structure and considering cancer invasion. Using agent-based modeling and a corresponding partial differential equation model, we demonstrate and prove mathematically a *tumor invasion paradox*: a larger cell death rate can lead to a faster invasion speed. We test this result on a generic hypothetical cancer with typical growth rates and typical treatment sensitivities. We find that the tumor invasion paradox may play a role for continuous and intermittent treatments, while it does not seem to be essential in fractionated treatments. It should be noted that no attempt was made to fit the model to a specific cancer, thus, our results are generic and theoretical.

## Introduction

The goal of definitive cancer treatment is to eradicate the disease. However, continuous therapies, often at maximum tolerable doses, fail to consider the intercellular dynamics of treatment response where competition, adaptation, and selection between treatment sensitive and resistant cells contribute to therapy failure (Gatenby 2009; Gatenby and Brown 2017). In fact, treatment may select for resistant phenotypes and/or eliminate other competing populations, possibly accelerating the emergence of resistant populations—a well-studied evolutionary phenomenon termed “competitive release” (Bolnick et al. 2010; Kotler and Brown 2020). In part to address this issue, clinical trials have explored intermittent on- and off-treatment protocols that provided comparable, and sometimes even prolonged, tumor control with significantly reduced treatment exposure (Bruchovsky et al. 2006; Zhang et al. 2017).

One possible explanation of treatment failure and aggressive tumor regrowth, both during and after therapy, are the so-called cancer stem cells (CSCs) (Gao et al. 2013; Brady-Nicholls et al. 2020). CSCs are often a minor sub-population of cancer cells that have traits comparable to normal tissue stem cells, including longevity, and the capacity to initiate and maintain a heterogeneous, aggressive tumor (Reya et al. 2001; Al-Hajj et al. 2003). Such cells were first identified in blood and blood cancers (Lapidot et al. 1994; Bonnet and Dick 1997), and later in many solid tumors (Clarke et al. 2006; Lee et al. 2016). The significant role of cancer stem cells in cancer development has recently been emphasized in the third “Hallmarks of Cancer” paper by Hanahan (2022). Extensive research is ongoing into the unique CSC properties (Lapidot et al. 1994). From this, many mathematical models were developed to elucidate the role and dynamics of tissue and cancer stem cells. (Enderling et al. 2009; Ry 2006; Hillen et al. 2013; Marciniak-Czochra et al. 2009; Solé et al. 2008; Agur et al. 2002).

In an agent-based model (ABM), Enderling and colleagues showed that CSCs compete with their non-stem tumor cells (TCs) progeny for resources and space. Further, they showed that selective killing TCs allows for competitive release of CSCs, accelerating tumor growth (Enderling et al. 2009). An integro-differential equation approach of the CSC-driven tumor dynamics showed that the *tumor growth paradox* arises under very general conditions (Enderling et al. 2009; Hillen et al. 2013). That is, cancers with a larger death rate for TCs—induced by conventional cytotoxic therapy, for example—yield tumors bigger than a comparable untreated tumor or tumors with lower death rates. A common conclusion from these models is that successful cancer therapies must eradicate all CSCs (Hillen et al. 2013; Dingli and Michor 2006).

Here, we place the tumor growth paradox into a spatial context. A *tumor invasion paradox* arises if increased cell death facilitates faster and further invasion into healthy tissue. In the context of radiation treatment, this phenomenon is also known as *radiation-induced invasion* (Maggiorella et al. 2005; Kargiotis et al. 2008; Jy 2018). The goal of the herein presented study is not to fit a model to a specific cancer or a specific treatment. Rather, we focus on a generic cancer model to understand the role of cancer stem cells on cancer invasion.

## Materials and Methods

To understand when the *tumor invasion paradox* is produced, we simulate CSC-driven tumor growth using two models, an agent-based model (ABM) that was formerly developed by Enderling et al. (2009) and a corresponding approach using partial differential equations (PDEs), as developed in Hillen et al. (2013). This way, we benefit from the advantages of both modeling types such as inclusion of stochasticity (ABM) and analytic tractability (PDE). Both models are based on the same assumptions:

1. CSCs are assumed to be immortal and can divide an unlimited number of times.
2. A CSC can divide symmetrically to produce two CSCs, or asymmetrically, to produce a CSC and a TC.
3. TCs have a limited life span with a positive death rate $\alpha > 0$.
4. TC have a limited proliferation capacity, and exclusively produce two TCs.
5. When a cell divides, one daughter cell remains in the parent cell’s location and the other daughter cell moves to unoccupied nearby space.
6. Cells are very small in comparison to the tissue and the tissue contains the cells. The domain $\varOmega$ represents the tissue.
7. Cells can randomly move in the domain $\varOmega$. However, movement is restricted by the availability of space. Thus, a cell can only move to a location with available space.

### The Agent-Based Model

As developed in Enderling et al. (2009), we simulate tumor growth and invasion in an empty two dimensional grid to examine intrinsic tumor dynamics without challenges from the environment. Each cancer cell occupies a single grid point within the rectangular computational lattice of $\omega =500 \times 50$ grid points. Cell behavior is updated at discrete time steps (or Monte Carlo steps, MCS) of $\varDelta t=1$ hr following its intrinsic properties and rules of engagement with adjacent cell agents. Cells are updated in a random order to avoid selection artifacts. At each MCS, a cell can migrate with probability $\mu =1$ (that is, cells can move one cell width each hour) and divide with probability $p=1/24$ (that is, cells divide on average one per day) if their remaining proliferative potential $\rho >1$ and at least one vacant adjacent grid point is available. If multiple lattice points in the cell’s neighborhood are vacant, a target position is selected at random.

**Fig. 1:**
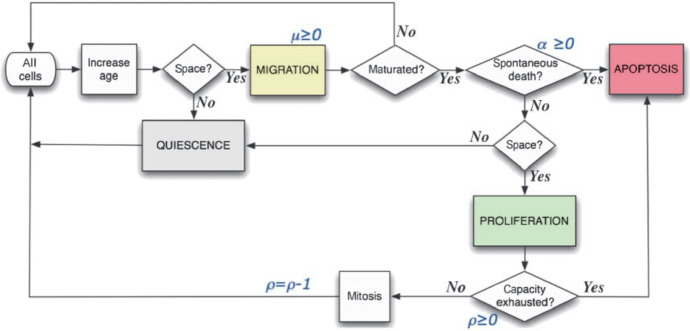
Agent-based model simulation decision flowchart. Reproduced from Enderling et al. (2009) (Color figure online)

TCs created by asymmetric CSC divisions are bestowed with a proliferation capacity of $\rho _{max}=15$. With each TC division, a decremented proliferation capacity of $\rho =\rho -1$ is inherited by both daughter cells. When $\rho =0$, the TC will undergo apoptosis and is removed from the simulation in the next proliferation attempt. Thus, each TC can generate a maximum population of $n = 2^{\rho _{max}}$ TCs. Additionally, at each MCS, TCs may be chosen at random to undergo spontaneous cell death. Herein, we explore different spontaneous cell death rates of $\alpha =0.01 - 0.35$ $\hbox {day}^{-1}$. In the simulation, each $\alpha$ value is divided by 24 to simulate spontaneous cell death per MCS. The flow diagram of the ABM is given in Fig. 1.

### The Continuum Model

We denote the CSC and TC population densities as *u*(*x*, *t*) and *v*(*x*, *t*), where $x\in \varOmega \subset {\mathbb {R}}$ is space and *t* is time. The total cancer cell density at location *x* and time *t* variable is then $n(x,t)=u(x,t)+v(x,t)$.

To formulate the above assumptions B1-B7 in a continuum framework, a spatially explicit integro-PDE for the spread and growth of cancer was developed in Hillen et al. (2013). An integral formulation was essential to include assumptions B5 and B7. In particular B5 lead to the use of so-called *birth-jump processes* (Hillen et al. 2014). Existence and uniqueness for this non-local model was shown in Borsi et al. (2014). Here we study a simplification that can be developed through a scaling analysis (Hillen et al. 2013). This reduction leads to a system of reaction-diffusion equations, which are the basis of our model here.

1 $$\begin{aligned}&u_{t} = Du_{xx} +p_s\gamma _u F(n)u\nonumber \\&v_{t} = Dv_{xx}+ (1-p_s)\gamma _u F(n)u+\gamma _v F(n)v - \alpha v \nonumber \\&n = u+v\, , \end{aligned}$$

where *D* denotes a diffusion coefficient, $p_s$ denotes the probability of symmetric CSC division, and *F*(*n*) is a monotonically decreasing function that describes space restrictions (Painter and Hillen 2002). The coefficients $\gamma _u$ and $\gamma _v$ are the growth rates for CSCs and TCs, respectively. When studying invasion waves on $\varOmega = (-\infty , \infty )$, we use the initial conditions

2 $$\begin{aligned} u(x,0) = u_{0}(x), \qquad v(x,0)=v_{0}(x), \end{aligned}$$

and boundary conditions

3 $$\begin{aligned} u(-\infty ,t)=1, \quad u(\infty ,t)=0. \end{aligned}$$

Further, for mathematical consistency, we assume

1. $u_{0}$, $v_{0}\in C(\varOmega )$, $u_{0}(x)$ and $v_{0}(x)$ are non-negative and $u_{0}(x)+v_{0}(x)\in [0,1].$
2. $F(n)\ge 0$ with $F(0)=1$ and $F(n)=0$ for $n\ge 1$ where *F* is continuous in the interval [0, 1].
3. $$F'(n)<0$$ for $$0\le n\le 1$$.

## The Tumor Invasion Paradox in the ABM

**Fig. 2:**
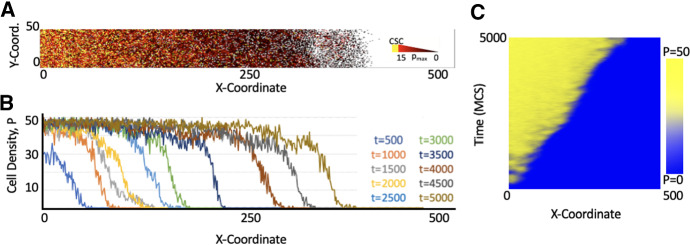
Agent-based simulation results. **A** Tumor population at t = 5000 MCS. CSCs are yellow, and TCs are visualized with a red-black gradient color based on their remaining proliferative potential, $\rho$. **B** Traveling wave pattern of population density $P \in {0,50}$ as a function of x-coordinate in the ABM lattice at different MCS marked in different colors. **C** Color-coded population density for each x-coordinate as a function of time visualizes the temporal gradient of invasion (Color figure online)

As shown in Fig. 2a, simulation of the ABM shows a continuous filling of the computational domain, $\varOmega$, with increasing MCSs. The initial CSCs at the left hand side of the domain is producing a majority of TCs that begin populating the initial CSC neighborhood. As TCs migrate or spontaneously die, CSCs become liberated to produce additional TCs or CSCs with low frequency. Over time, the “oldest” area of the tumor (lower *x*-coordinates) has the highest proportion of CSC, whereas the rightmost area recently populated by cancer cells for the first time (larger *x*-coordinates) harbors predominantly non-stem TCs with highly limited remaining proliferative potential, $\rho$ (Fig. 2a). Quantitative analysis of cell density over the *x*-coordinate in $\varOmega$ shows the highest density of cells towards the left hand side of the domain, the location of the initial CSCs, with declining cell densities deeper into the domain. After the vertical domain reached its carrying capacity at low *x*-coordinates, a traveling wave pattern of tumor expansion emerges (Fig. 2b, c).

**Fig. 3:**
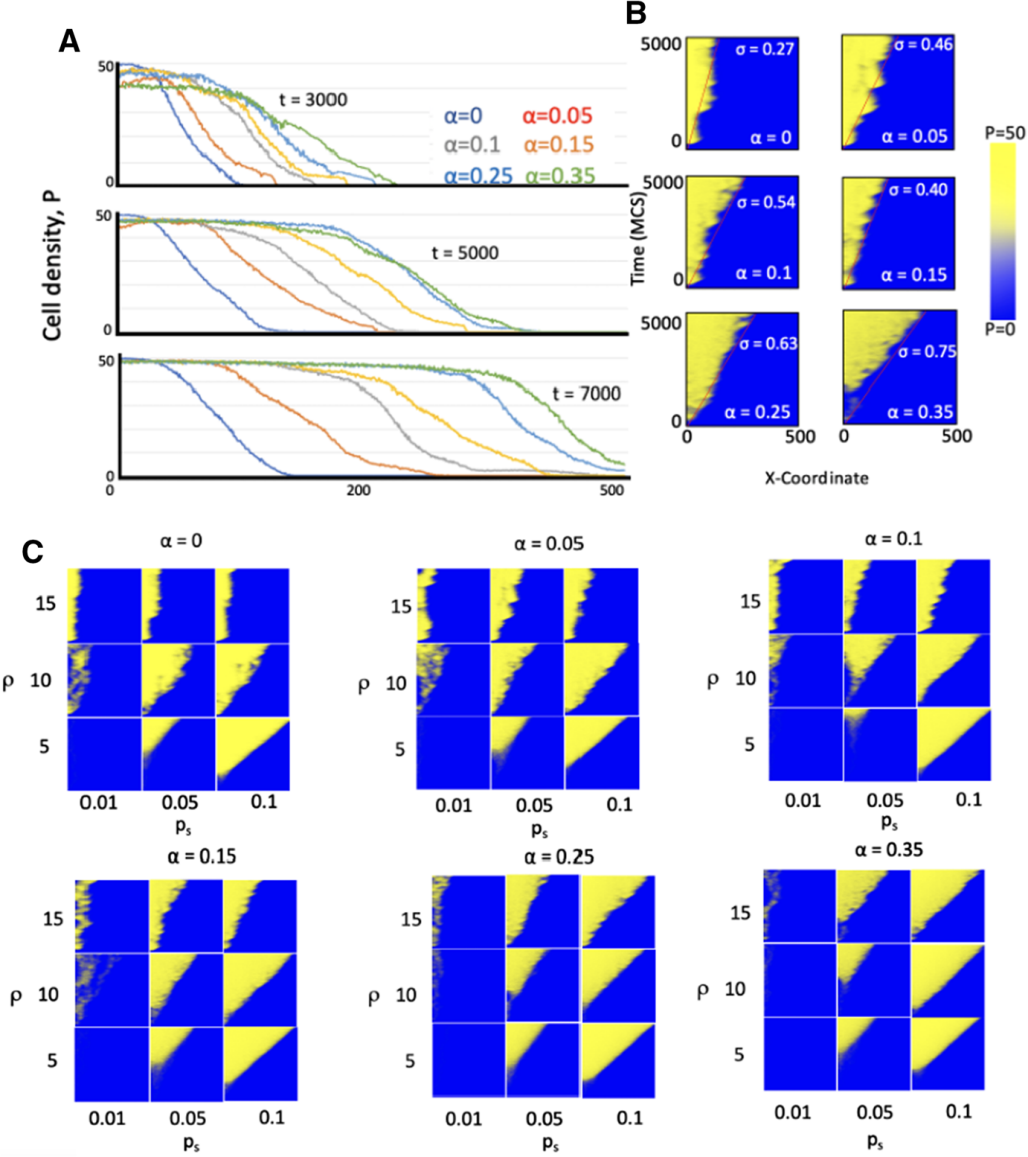
**A** Tumor growth for increasing values of $\alpha$ at times t = 3000, t = 5000, and t = 7000 MCS. As time increases, the separation between individual curves increases, showing tumors with larger values of $\alpha$ have faster invasion speeds. **B** Heat maps compiled for the invasions of tumors with various death probabilities. The interface between yellow and blue is steeper for the lower death probabilities and as the value of $\alpha$ increases, the yellow reaches further into x domain over time. Invasion speeds as $\alpha$ ranges from $\alpha = 0$ to $\alpha = 0.35$ are 0.0058 pixels/MCS, 0.0156 pixels/MCS, 0.0336 pixels/MCS, 0.0468 pixels/MCS, 0.0986 pixels/MCS, 0.1122 pixels/MCS. **C** Parameter sensitivity analysis showing gradient of the wave front over all MCS for different proliferation capacities, $\rho$, and CSC division rates, $p_s$ (Color figure online)

We then compared the evolution of the cell density as a function of space (*x*-coordinate) in $\varOmega$ for different spontaneous cell death rates $\alpha = (0, 0.05, 0.1, 0.15, 0.25, 0.35)$ $\hbox {day}^{-1}$ at different MCS. Spontaneous cell death is very common in carcinomas that arise from continuously renewing epithelial tissues. Experimental animal tumors have cell loss factors of up to 92$\%$. Human tumors have an average cell loss in excess of 50$\%$, with the median estimate of the cell loss factor being 77$\%$ (Hall and Giaccia 2012). For the purpose of this study, we did not aim to calibrate any model parameters, but rather compare tumor invasion dynamics as parameter values change. Lower $\alpha$ values yield initially higher densities for lower *x*-coordinates in the ABM, but higher $\alpha$ lead to further invasion into the domain, arguably due to competitive release of CSCs in line with previous observations (Enderling et al. 2009). While all simulations demonstrated a traveling wave-like invasion of the spatial domain, higher cell death rates $\alpha$ led to faster invasion speeds (Fig. 3a). Analysis of the speed of the wave front, $\sigma$, over all MCS confirmed higher invasion speeds for higher spontaneous cell death rates (Fig. 3b), showing the *tumor invasion paradox*. The wave front evolution over time for different proliferation capacities, $\rho$, CSC symmetric division probabilities, $p_s$, and death rates, $\alpha$, is shown in Fig. 3c. We note that the tumor invasion paradox disappears for larger values of the CSC self-renewal probability $p_s$.

## The Invasion Paradox in the Continuum Model

We have clearly demonstrated the tumor invasion paradox in our ABM simulations. Here we employ the corresponding PDE formulation (1) and prove mathematically that the invasion paradox exists for certain parameter ranges. We will use a travelling wave analysis and show in Theorem 1 that the invasion speed monotonically increases with the TC death rate $\alpha$.

For simplicity, we assume that the cell cycle times for CSCs and TCs are the same, with $\gamma _u = \gamma _v = 1.$ For our analysis we employ a scaling argument based on the fact that the probability of CSC self-renewal is small, i.e. we assume that $p_s=\varepsilon$, where $\varepsilon >0$ denotes a small dimensionless quantity. Further, we assume that diffusion (i.e. cell movement) is slow and on the same scale $D=\varepsilon d$, where *d* is order one. Further, the cells can reproduce quickly with reproduction occurring on the time scale of *O*(1).

With the above assumptions, (1) becomes

4 $$\begin{aligned}&u_{t} =\varepsilon du_{xx} +\varepsilon F(n)u,\nonumber \\&v_{t} = \varepsilon dv_{xx}+ (1-\varepsilon ) F(n)u+ F(n)v - \alpha v. \end{aligned}$$

As $\varepsilon$ is a small parameter, there is a separation of time scales, where cell movement is slow and cell division is fast. Thus, we apply geometric singular perturbation theory (Hek 2010) which allows us to separate (4) into a fast system and a slow system.

If $\varepsilon \rightarrow 0,$ we obtain the fast system

5 $$\begin{aligned}&u_{t} = 0\nonumber \\&v_{t} = F(n)u+ F(n)v - \alpha v, \end{aligned}$$

where the nontrivial steady states define the slow manifold

6 $$\begin{aligned} M:=\{(u,v):\alpha v = F(u+v)(u+v)\}. \end{aligned}$$

**Fig. 4:**
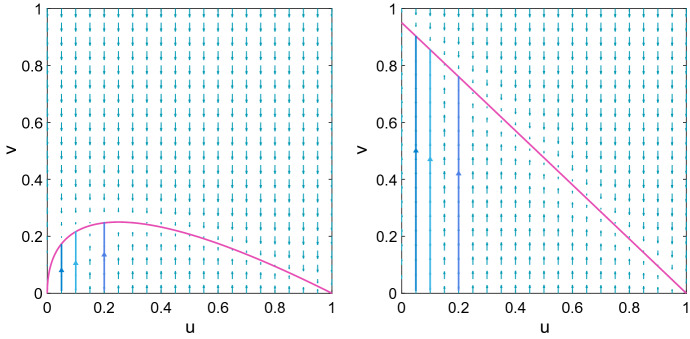
Phase portraits with $\alpha =1$ (left) and $\alpha =0.05$ (right). The solution trajectories (shown by blue lines) converge to the slow manifold (shown in pink) (Color figure online)

The fast system has trivial flow, as *u* is constant and *v* settles on the slow manifold. In Fig. 4 we show the slow manifold for two cases of $\alpha$, $\alpha =1$ and $\alpha =0.05$, as well as a few orbits of the fast system in blue. It is evident that the fast solutions converge to the slow manifold *M*. Thus, the essential dynamics of the full system occur on the slow manifold *M* and are described by the slow system.

To obtain the slow system, we first re-scale (4) by setting $\tau = \varepsilon t$ to obtain

7 $$\begin{aligned}&\varepsilon u_{\tau } = \varepsilon du_{xx} +\varepsilon F(n)u \nonumber \\&\varepsilon v_{\tau } = \varepsilon dv_{xx}+ (1-\varepsilon ) F(n)u+ F(n)v - \alpha v. \end{aligned}$$

Then, letting $\varepsilon \rightarrow 0$, we obtain the slow system

8 $$\begin{aligned}&u_{\tau } = d u_{xx} + F(u+v)u \nonumber \\&0= F(u+v)u+ F(u+v)v - \alpha v. \end{aligned}$$

The second equation shows that the dynamics occur on the slow manifold *M*, while the first equation describes the dynamics on *M*.

### Properties of the Slow Manifold

The slow manifold, *M*, has been studied in detail by Hillen et al. (2013), and we summarize its main properties in the following Lemma. We sketch the important arguments in a little proof.

#### Lemma 1

Assume $\alpha >0$. The slow manifold *M* from (6) has the following properties

1. The slow manifold can be written as a graph $v_{\alpha }(u)$ for $0\le u\le 1.$
2. The graph $v_\alpha (u)$ is differentiable and has the derivative
  9 $$\begin{aligned} v_\alpha '(u) = \frac{F'(n(u)) n(u) + F(n(u))}{\alpha - F'(n(u)) n(u) - F(n(u))}, \end{aligned}$$
  where $n(u) = u + v_{\alpha }(u)$ denotes the total cell density on *M*.
3. If $\alpha <1$, then there exists a unique $v_{\alpha }^*>0$ with $F(v_{\alpha }^*)=\alpha$ and the slow manifold connects to $(0,v_{\alpha }^*)$.
  If $\alpha \ge 1$ then the slow manifold connects to (0, 0). In this case $v_{\alpha }^*=0$.
4. $$v_{\alpha }(u)>0$$ for $$0<u<1$$ and
  $$\begin{aligned} \lim _{u \rightarrow 0} v_{\alpha }(u) = v_{\alpha }^*, \qquad \lim _{u \rightarrow 1} v_{\alpha }(u) = 0. \end{aligned}$$
5. The slope of the slow manifold at the point $(0,v_{\alpha }^*)$ is given by
  $$\begin{aligned} v_{\alpha }'(0)= -\frac{\alpha +F'(v_{\alpha }^*)v_{\alpha }^*}{F'(v_{\alpha }^*)v_{\alpha }^*}. \end{aligned}$$

#### Proof

Assume $$\alpha >0$$

1. The solution of $v_{\alpha }(u)$ is given implicitly by
  10 $$\begin{aligned} (\alpha -F(u+v))v=F(u+v)u. \end{aligned}$$
  It was shown in Lemma 3.2 in Hillen et al. (2013) that $v_{\alpha }(u)$ is a graph.
2. The derivative can be computed from (10) by implicit differentiation.
3. Item 3 follows by finding the steady states of the slow system. Letting $u\rightarrow 0$, the first equation in (8) becomes 0 and the second equation, which defines *M*, becomes
  $$\begin{aligned} 0 = F(v)v-\alpha v \end{aligned}$$
  which is equivalent to
  $$\begin{aligned} (\alpha - F(v))v=0. \end{aligned}$$
  Since *F*(*n*) is decreasing, if $\alpha > F(0)$, then *v* must be 0 to satisfy the above equation. Thus, the slow manifold connects to (0, 0). If $0<\alpha <F(0)$, then there exists a unique $v_{\alpha }^*>0$ such that $F(v_{\alpha }^*)= \alpha$, since *F* is continuous on this interval. In this case, the slow manifold connects to $(0,v_{\alpha }^*)$.
4. Item 4 follows from the definition of $v_{\alpha }^*$ in item 3 above and from the definition of a maximum capacity of $n=1$ in assumption A2.
5. Item 5 follows from the formula computed in the proof of Lemma 3.2 in Hillen et al. (2013) by setting $k(p)=F(n)$.

$$\square $$

An observation that is central to the tumor growth paradox is the following Lemma, which shows that the slow manifold for large $\alpha$ is situated lower in phase space.

#### Lemma 2

For each $u\in (0,1)$, $\alpha _{1}>\alpha _{2}$ implies $v_{\alpha _{1}}(u)<v_{\alpha _{2}}(u)$.

#### Proof

Rewrite (10) as

$$\begin{aligned} \frac{1}{v} = \frac{\alpha }{F(u+v)u}-\frac{1}{u}. \end{aligned}$$

The right hand side is increasing in $\alpha$ and increasing in *v*, and $F(u+v)$ is monotonically decreasing and bounded above by 1. Thus, if $\alpha$ is increased, *v* must decrease. $\square$

Finally, we show that the total population $n=u+v$ is increasing along the slow manifold *M* as a function of *u*.

#### Lemma 3

Assume $a>0$ and write the total population on *M* as

$$\begin{aligned} n(u)= u + v_{\alpha }(u), \qquad u\in [0,1]. \end{aligned}$$

Then $$n'(u)>0$$.

#### Proof

We first compute a condition when the slow manifold *M* is tangential to the population level lines, i.e. when $v'_\alpha (u) =-1$. By formula (9) the previous equation is equivalent to

$$\begin{aligned} F'(n(u)) n(u) + F (n(u)) = -\alpha + F'(n(u)) n(u) + F(n(u)), \end{aligned}$$

which is true only for $\alpha =0$.

Next we show that $v'_\alpha (0)>-1$, which then implies $v'_\alpha (u)>-1$ for all $u\in [0,1]$. From (9) we find

$$\begin{aligned} v'_\alpha (0) = \frac{F'(v_{\alpha }^*) v_{\alpha }^* + F(v_{\alpha }^*) }{\alpha - F'(v_{\alpha }^*) v_{\alpha }^* - F(v_{\alpha }^*)}. \end{aligned}$$

In the case of $\alpha > 1$ we have from Lemma 1 that $v_{\alpha }^*=0$ and $F(0)=1$, hence

$$\begin{aligned} v'_\alpha (0) = \frac{1}{\alpha -1} >-1. \end{aligned}$$

In the case of $\alpha <1$ we have $v_{\alpha }^*>0$ and $F(v_{\alpha }^*) = \alpha$. In that case

$$\begin{aligned} v'_\alpha (0) = \frac{F'(v_{\alpha }^*) v_{\alpha }^*+\alpha }{\alpha - F'(v_{\alpha }^*) v_{\alpha }^* - \alpha } = -\left( 1 + \frac{\alpha }{F'(v_{\alpha }^*) v_{\alpha }^*} \right) \; > \; -1, \end{aligned}$$

since *F* is decreasing.

The case $\alpha =1$ is a limiting case and needs extra attention. For $\alpha =1$ we consider the limit as $u\rightarrow 0$ of

$$\begin{aligned} \lim _{u\rightarrow 0} v_1'(u) = \lim _{u\rightarrow 0} \; \frac{F'(n(u)) n(u) + F(n(u))}{1 - F'(n(u)) n(u) - F(n(u))} = +\infty ,\end{aligned}$$

as the numerator converges to 1 and the denominator to $+0$.

Taken all together, we find that since $n'(u)=1+v'_{\alpha }(u)$, then $v'_{\alpha }(u)>-1$ implies $n'(u)>0$.

$$\square $$

### Travelling Waves on the Slow Manifold

On the slow manifold, our problem (8) becomes

11 $$\begin{aligned} u_{\tau } = d u_{xx} + F(u+v_{\alpha }(u))u \end{aligned}$$

which looks like a Fisher–KPP-type equation. To be specific we define a Fisher–KPP-type equation as follows.

#### Definition 1

We call a reaction-diffusion equation of the form

$$\begin{aligned} u_t = d u_{xx} + f(u) \end{aligned}$$

to be of Fisher–KPP type if there exists a carrying capacity $K>0$ and

$$\begin{aligned} i)&\ \ f(0)=0, \quad{} & {} f(K)=0,\\ ii)&\ \ f(u)>0 \quad{} & {} \text {for all } \ 0<u<K,\\ iii)&\ \ f'(0)>0, \quad{} & {} f'(K)<0 . \end{aligned}$$

This type of equation is often also called the *monostable case* (De Vries et al. 2006, p. 113-114).

We show that this is indeed the case for (11).

#### Lemma 4

Under the assumptions A2 and A3, system (11) is a Fisher–KPP-type equation.

#### Proof

We note that $K=1$ as 1 denotes the maximum capacity. Further,

$$\begin{aligned} f(u) = F(u+v_{\alpha }(u))u. \end{aligned}$$

Then

12 $$\begin{aligned} f(0)= F(v_{\alpha }(0))0=0 \quad \text {and} \quad f(1)=F(1+v_{\alpha }(1))1=0, \end{aligned}$$

which follows from the definition of *F* as $v_{\alpha }(1)=0$ and $F(1)=0$. Further $f(u)>0$ since $F>0$ for $u\in (0,1)$, by definition. Thus

$$\begin{aligned} f'(u) = F'(u+v_{\alpha }(u))(1+v_{\alpha }'(u))u+F(u+v_{\alpha }(u)) \end{aligned}$$

and

$$\begin{aligned} f'(0) = F(v_{\alpha }(0))=F(v_{\alpha }^*). \end{aligned}$$

Since $0\le v_{\alpha }^*< 1$ by definition, $F(v_{\alpha }^*)>F(1)=0$ as *F* is monotonically decreasing. Hence,

13 $$\begin{aligned} f'(0)>0 \quad \text {and} \quad f'(1)=F'(1+v_{\alpha }(1))(1+v_{\alpha }'(1)). \end{aligned}$$

Since *F* is monotonically decreasing we require

$$\begin{aligned} 1+v_{\alpha }'(1)>0, \end{aligned}$$

which holds by Lemma 3. $\square$

### Travelling Wave Analysis

Because we showed that (11) is a Fisher–KPP-type equation on *M*, we can consider travelling waves of the form

$$\begin{aligned} u(x, \tau ) = \phi (x-\sigma \tau ), \end{aligned}$$

where $\phi$ is the function that defines the shape of the wave and $\sigma$ is the invasion speed. The boundary conditions are

$$\begin{aligned} \phi (-\infty )=1, \qquad \phi (\infty )=0, \end{aligned}$$

meaning that the tumor is at its carrying capacity at $x \rightarrow -\infty$ and has not arrived at $x \rightarrow \infty$ (De Vries et al. 2006, p. 112). From this solution, we calculate the invasion speed of the tumor.

Before we give the result, we first define what we mean by an invasion initial condition.

#### Definition 2

(*Invasion initial conditions*) Consider a monotonically decreasing $u_{0}(x)$ such that

$$\begin{aligned} \lim _{x \rightarrow -\infty } u_{0}(x)=1 \end{aligned}$$

and that there exists a threshold value $x^*$ such that

$$\begin{aligned} u_{0}(x^*)=0 \quad \text { for all } x>x^*. \end{aligned}$$

Under these conditions, $u_{0}(x)$ defines an *invasion initial condition*.

#### Theorem 1

Assume that $\alpha >0$ and that the assumptions A1–A3 hold. Let $v_{\alpha }(u)$ denote the parameterization of *v* on the slow manifold *M* given implicitly by (6). Let $u(x,\tau )$ be a solution of (11) with invasion initial conditions.

(i) If $a\ge 1$, then $u(x,\tau )$ converges to a travelling wave with a minimum wave speed
  $$\begin{aligned} \sigma ^*=2\sqrt{d}. \end{aligned}$$
(ii) If $\alpha <1$, then $u(x,\tau )$ converges to a travelling wave with a minimum wave speed
  $$\begin{aligned} \sigma ^* = 2\sqrt{d\alpha }. \end{aligned}$$
  These cases connect continuously for $\alpha \rightarrow 1$.

#### Proof

Again we use

$$\begin{aligned} f(u):= F(u+v_{\alpha }(u)) u. \end{aligned}$$

If system (11) is linearly determined (nonlinear spread rates are equal to the linearized rates at the leading edge of the wave) (Castillo-Chavez et al. 2013, p. 1), it follows that the minimum wave speed will be $\sigma ^*=2\sqrt{df'(0)}$ (Al-Kiffai and Crooks 2016, Proposition 2.2).

A sufficient condition for linear determinacy (Al-Kiffai and Crooks 2016, Proposition 2.2) is

$$\begin{aligned} f(u)\le f'(0)u. \end{aligned}$$

Since $f'(u) = F'(u+v_{\alpha }(u))(1+v_{\alpha }'(u))u+F(u+v_{\alpha }(u))$, we have that

$$\begin{aligned} f'(0)=F(v_{\alpha }(0)). \end{aligned}$$

By *3* in Lemma 1, $F(v_{\alpha }(0))=F(v_{\alpha }^*)$.

Thus, the condition for linear determinacy becomes

$$\begin{aligned} F(u+v_{\alpha }(u))u\le F(v_{\alpha }^*)u. \end{aligned}$$

Noting that *F* is monotonically decreasing, the above condition is equivalent to

14 $$\begin{aligned} u+v_{\alpha }(u)\ge v_{\alpha }^*, \end{aligned}$$

or in other words $n(u)\ge v_{\alpha }^*$. At $u=0$ we have equality, and in Lemma 3 we had shown that $n'(u)>0$. Hence (14) is indeed satisfied. Thus, the system is linearly determined and it follows that an invasion initial condition converges to a travelling wave with minimal speed

$$\begin{aligned} \sigma ^*=2\sqrt{df'(0)}=2\sqrt{dF(v_{\alpha }^*)}. \end{aligned}$$

In the case $\alpha \ge 1$ we have $v^*_a =0$ and $F(0)=1$, which is the first case in the Theorem. For $\alpha <1$ we have $v^*_\alpha >0$ and $F(v_{\alpha }^*)=\alpha$ and we get the second case. $\square$

#### Remark

1. This result implies that for $\alpha <1$, we have a *tumor invasion paradox* because the invasion speed, $\sigma ^*$, is directly proportional to $\sqrt{\alpha }.$ Hence, a larger death rate for TCs leads to a faster tumor invasion if conditions in *ii)* are satisfied. Otherwise, if conditions for *i)* are satisfied, the tumor spreads at a constant invasion speed regardless of the death rate.
2. If we look at the dimensions of the corresponding terms, then it should be noted that we set the reproduction rates $\gamma _u=\gamma _v=1$, and these carry a unit of (time)$^{-1}$. By setting these equal to 1, we effectively transfer a unit (time)$^{-1}$ to the functions *F*. Then the equation $F(0)=1$ carries a unit (time)$^{-1}$, such that the travelling wave speed is indeed a speed.

### Numerical Simulations of the Continuum Model

We show that the wave speeds in Theorem 1 coincide with numerical wave speeds. To show this, we solve (11) numerically by choosing $F=(1-n)^+$, where *F* is a monotonically decreasing function on [0, 1], $F(0)=1$, $F(1)=0$, and the index $+$ indicates the non-negative part.

We focus on studying the death rates $\alpha \in \{ 0.05, 0.5, 1,5\}$. This parameter range is chosen to include the change in behavior around $\alpha =1$, as stated in our main result in Theorem 1. In order to simulate system (11), we choose $d=1$ as it is convenient for our choice of simulation time. We solve system (11) using the solver *pdepe* in MATLAB.

**Fig. 5:**
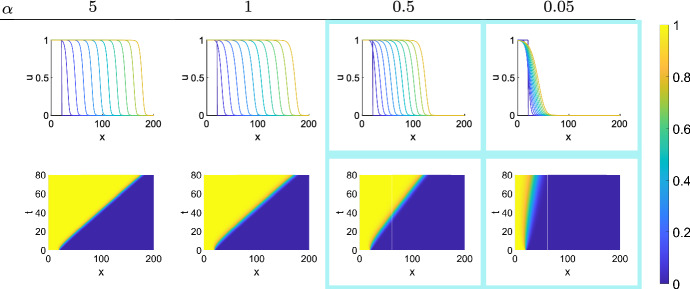
Travelling wave solutions of (11) at $t=0,8,16,\ldots 80$ (top row) and top view of the solution surface of system (11) (bottom row). The value of $\alpha$ used in each simulation is shown in the column headings. The highlighted regions indicate cases where solutions undergo the invasion paradox (Color figure online)

The results of the simulations are summarized in Fig. 5. We represent an infinite domain by setting initial density $u_{0}(x)=1$ for $x\le 20$ and $u_{0}(x)=0$ for $x>20$ which allows the left bound to represent $-\infty$ and the right bound to represent $\infty .$ We chose a simulation of $t=80$ because it captures the main dynamics of the system.

Figure 5 shows both the top view of the solution surface and wave profile solutions at particular time points for system (11) for varying $\alpha$. It takes some time for the initial solution to converge to a travelling wave solution as the discontinuous initial conditions requires time to diffuse and become a wave that retains its shape for all time. The smaller the $\alpha$, the longer it takes to establish a travelling wave profile. The highlighted sections in Fig. 5 indicate the cases where the invasion paradox occurs (i.e. where $\alpha <1$). As the death rate decreases, the speed of the travelling waves $\sigma$ also decreases which is visualized by examining the diagonal “line” that is forms the boundary between high density and low density regions. The slope of this line represents the travelling wave speed.

We compare the numerical and theoretical wave speeds in Table 1. The numerical wave speeds were calculated by taking the average distance travelled by the wave profile plots in Fig. 5 and dividing it by the time it took to travel that distance. We perform these calculations after the wave has been established. The invasion paradox is illustrated clearly in Table 1 in bold face, where the speeds decrease as $\alpha$ decreases. When $\alpha \ge 1$, there is no invasion paradox; hence, the wave speed is the same for all $\alpha >1$.

**Table 1:** Comparison of average numerical wave speeds and theoretical wave speeds for $d=1$

$\alpha$	Numerical speed	Theoretical speed
5	*2.0340*	*2.0000*
1	*2.0044*	*2.0000*
0.8	**1.7757**	**1.7889**
0.5	**1.4036**	**1.4142**
0.05	**0.4111**	**0.4472**

In the italic regions, the tumor growth paradox is not present, while in the bold regions, the tumor growth paradox is present

We also examined the invasion paradox for four other volume-limitation functions, *F*(*n*), including

15 $$\begin{aligned} F(n) \in \left\{ 1-n,\ 1-n^4, \ \frac{1}{e-1}(e^{-(n-1)}-1),\cos \left( \frac{\pi n}{2}\right) \right\} ^+. \end{aligned}$$

All of these functions satisfy the assumptions A2 and A3. Each function *F* is nonnegative, continuous, and monotonically decreasing in the interval [0, 1]. Further, $F(0)=1$ and $F(1)=0$ hold for each function. In all cases we find the invasion paradox, very similar to those in Fig. 5. As the results for the volume-limitation functions given by (15) are consistent with those for $F=1-n$, the results and simulations for the additional forms of *F* are not shown here for the sake of brevity.

## Implications on Cancer Treatment

We have now established that the tumor invasion paradox is a robust phenomenon in stem-cell driven tumors, which arises for a large range of parameter values. Here, we would like to use our models to explore its relevance in cancer treatment. As mentioned before, it is not our goal to make specific treatment predictions for a specific cancer. To do that, we would need a fully calibrated model. Instead, we employ our models with typical treatment parameters and investigate its impact on cancer treatment.

### Intermittent Therapy as seen from the ABM

**Fig. 6:**
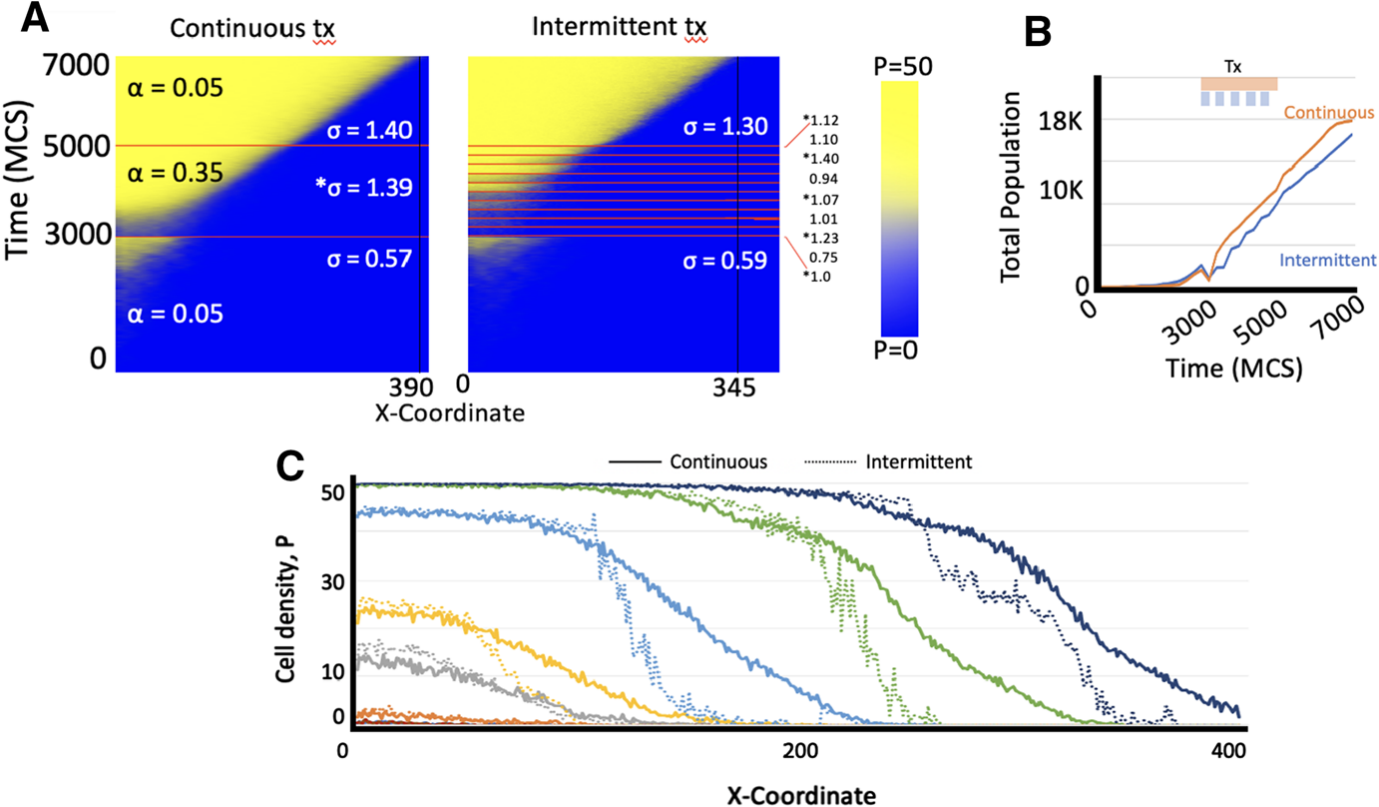
Agent-based simulation results. **A** Heat maps for tumor invasiveness under continuous and intermittent treatments. Non-treatment cell death is $\alpha = 0.05$ and treatment death is $\alpha = 0.35$. In continuous treatment, the simulated tumor underwent 3000 MCS without treatment, 2000 MCS with treatment, then 2000 MCS again without treatment. In intermittent treatment, the simulated tumor underwent 3000 MCS without treatment, 2000 MCS with treatment in intervals of 200 MCS (indicated by *), then 2000 MCS again without treatment. **B** Total growth of tumors under continuous and intermittent treatment. Logarithmic growth and overlapping curves until 3000 MCS when separation occurs. Total population of tumor under continuous treatment is consistently larger than that of intermittent treatment after 3000 MCS. **C** Total tumor populations at each x position in domain for tumors under continuous and intermittent treatments at time steps $t = 1000$, $t = 2000$, $t = 3000$, $t = 4000$, $t = 5000$, $t = 6000$, and $t = 7000$ MCS. Corresponding colors indicate corresponding time steps. After 3000 MCS, continuous treatment tumors consistently populated further into the domain than intermittent treatment tumors at same time steps (Color figure online)

To test if the invasion paradox is sustained under treatment-like scenarios, we simulate tumor growth with a fixed low spontaneous cell death rate, $\alpha =0.05$, for $t=3000$ MCS. From $t=3000-5000$ MCS we simulate cancer therapy with a higher death rate $\alpha =0.35$, before simulating post-therapy growth dynamics until $t=7000$ MCS. We compare continuous therapy with a total on-treatment time of 2000 MCS with an intermittent treatment that toggles five on-treatment periods with $\varDelta t=200$ MCS with treatment holidays (total treatment time of 1000 MCS that delivers half of the dose compared to continuous therapy). Before therapy, both simulations have comparable numerical speed of the wave front ($\sigma _{\hbox {{continuous}}}=0.57$ vs. $\sigma _{\hbox {{intermittent}}}=0.59$; Fig. 6a). During continuous therapy, $\sigma _{\hbox {{continuous}}}$ increases to $\sigma _{\hbox {{continuous}}}=1.39$ and remains increased post-therapy. During each intermittent on-treatment, $\sigma _{\hbox {{intermittent}}}>1$ with lower $\sigma _{\hbox {{intermittent}}}$ during treatment holidays (Fig. 6a). Analysis of the total population shows that continuous therapy yields larger total cancer cell populations compared to intermittent therapy - a manifestation of the the tumor growth paradox (Fig. 6b)—as well as deeper tissue invasions - the tumor invasion paradox (Fig. 6c).

### Radiation Treatment as seen from the PDE

We now study how the model (4) behaves when treatment is added. To study the behavior, we choose to work with $F(n)=(1-n)^+$ as this is the simplest choice for the volume function *F* and, as previously noted, the more complicated functions behave in a similar fashion. We assume that the treatment increases the death rate of TCs, and that CSCs are more resistant to treatment. For simplicity, we assume that the CSC sensitivity to radiation is small and of the scale $\varepsilon$. Under these assumptions, the model becomes

16 $$\begin{aligned}&u_{t} =\varepsilon du_{xx} +\varepsilon F(n)u - \varepsilon {\hat{b}} u\nonumber \\&v_{t} = \varepsilon dv_{xx}+ (1-\varepsilon ) F(n)u+ F(n)v - {\hat{\alpha }} v, \end{aligned}$$

where

$$\begin{aligned} {\hat{\alpha }}(t)= {\left\{ \begin{array}{ll} \alpha + T(t) &{} \ \text {when treatment is applied}\\ \alpha &{} \ \text {otherwise} \end{array}\right. } \end{aligned}$$

and

$$\begin{aligned} {\hat{b}}(t) = {\left\{ \begin{array}{ll} \delta T(t) &{} \qquad \text {when treatment is applied}\\ 0 &{} \qquad \text {otherwise} \end{array}\right. }. \end{aligned}$$

Here, $\delta$ is an additional parameter that scales *T* in order to account for the CSC treatment resistance. The parameter $\delta$ allows us to vary the effect on the stem cells without changing the scaling $\varepsilon$. Applying geometric singular perturbation theory to (16), we obtain the following slow system

17 $$\begin{aligned}&u_{\tau } =du_{xx} + F(u+v)u - {\hat{b}} u\nonumber \\&0 = F(u+v)(u+v) - {\hat{\alpha }} v. \end{aligned}$$

Similar to the ABM, we study continuous and intermittent therapy. To model the radiation treatments, we choose $\alpha =0.012$ $\hbox {day}^{-1}$ (Bennett et al. 2021). We use diffusion coefficients and radiosensitivities that are often used for prostate cancer modeling and glioma modeling. The diffusion coefficients are $d_p=8.64\times 10^{-4}$ $\hbox {mm}^2$ $\hbox {day}^{-1}$ (prostate case) (Jackson 2004) and $d_g=0.65$ $\hbox {mm}^2$ $\hbox {day}^{-1}$ (glioma case) (Swanson et al. 2000) and the radiosensitivity coefficients are $\alpha _p =0.15$ $\hbox {Gy}^{-1}$, $\beta _p = 0.048$ $\hbox {Gy}^{-2}$ (prostate case) and $\alpha _g =0.11$ $\hbox {Gy}^{-1}$, $\beta _g = 0.019$ $\hbox {Gy}^{-2}$ (glioma case) (Van Leeuwen et al. 2018). The treatment function, *T*(*t*), is modeled by a hazard function approach, which approximates the cell death rate caused by treatment and varies depending on the type of therapy (Gong et al. 2013).

#### Continuous and Intermittent Radiation

In our first case, we simulate a situation of continuous radiation. One example of such a treatment is brachytherapy for prostate cancer (Nag et al. 1999). To simulate the treatment, we use the parameters for prostate cancer from above and the corresponding hazard function from Gong et al. (2013)

18 $$\begin{aligned} T(t) = \alpha _p R_{0} e^{-\lambda t} + 2\beta _p \frac{R_{0}^2}{\lambda }(e^{-2\lambda t + \lambda \omega } - e^{-2 \lambda t}), \end{aligned}$$

where $R_0$ is the initial treatment dose rate, $\lambda$ is the decay rate of the therapy, and $\omega$ is the size of the effective interaction window of independent treatment events compounding. This form is essentially equivalent to the widely used Leah-Catchside protraction factor in radiotherapy (Gong et al. 2013), except that the above form is more tractable. Here, we use the parameters $R_0=5.71$ Gy $\hbox {day}^{-1}$, $\lambda =0.0408$ $\hbox {day}^{-1}$, and $\omega =1/90$ $\hbox {day}^{-1}$ (Gong et al. 2013) to examine a typical brachytherapy treatment. We also set $R_0=50$ Gy $\hbox {day}^{-1}$ to examine an extreme treatment with a high dose rate.

**Fig. 7:**
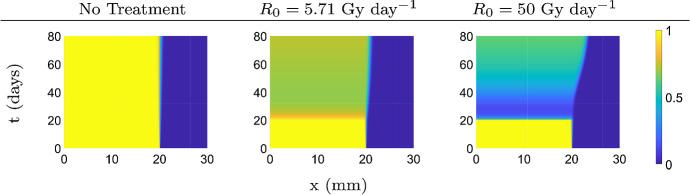
Top view of the solution surfaces of system (17) with $\delta =0.3$ under therapy. Treatment begins on day 20 and the simulation ends on day 80 (Color figure online)

With these parameters, we simulate the model to obtain the plots given in Fig. 7. The first image shows the cancer development without any treatment. The tumor persists and does not spread in any significant way. In the next two images, therapy is applied on day 20, with the low dose rate of $R_0=5.71$ Gy $\hbox {day}^{-1}$ in the middle figure and a large dose rate $R_0=50$ Gy $\hbox {day}^{-1}$ in the right figure. We see that treatment has an effect on the spatial spread of the tumor region, being significantly increased for large $R_0$. However, at the same time the tumor burden goes down due to treatment efficacy. Hence we observe a trade-off between successful treatment versus extended spread.

**Fig. 8:**
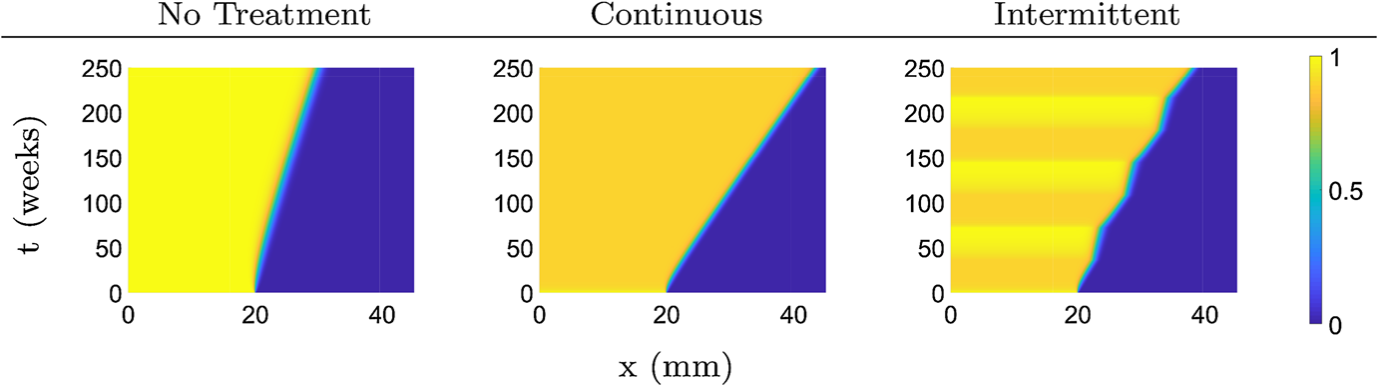
Top view of the solution surfaces of system (17) with $\delta = 0.1$ under continuous and intermittent treatments. Treatment type in each simulation is as indicated by the column headings (Color figure online)

In Fig. 8, we use the same model parameters as before and change the treatment schedule to intermittent, as outlined by Brady-Nicholls et al. (2020). In the intermittent case, the treatment begins after four weeks, and is applied continuously for 36 weeks on followed by 36 weeks off, repeatedly. We clearly see the increased invasion during treatment time, and also note that continuous treatment leads to the highest tumor spread on the final week $t=250$.

#### Fractionated Treatment

To examine how the system (17) behaves under fractionated therapy, we adopt the hazard function (Gong et al. 2013) to

19 $$\begin{aligned} T(t)=(\alpha _g + \beta _g d) d(t), \end{aligned}$$

where *d* denotes the dose rate per fraction and the notation *d*(*t*) is used to indicate that this term is only used during the actual treatment window, otherwise it is zero. In this case we take the radiosensitivities of the glioma case $\alpha _g =0.11$ $\hbox {Gy}^{-1}$, $\beta _g = 0.019$ $\hbox {Gy}^{-2}$, and a diffusion rate of $d_g=0.65$ $\hbox {mm}^2$ $\hbox {day}^{-1}$.

We consider three types of fractionation schedules. In the first case we consider 20 days on followed by 20 days off repeated for 60 days of treatment with a dose rate of 1.5 Gy $\hbox {day}^{-1}$. The second case considers 2 days on and off for 60 days of treatment with a dose rate of 2 Gy $\hbox {day}^{-1}$. The third case is a situation of short 10-minute radiation bursts, once per day for five week days per week, with a dose rate of 2 Gy (10 min)$^{-1}$. The last case is a typical fractionated treatment schedule, and in our case this treatment lasts for 6 weeks. In all cases we start the treatment on day 20 and consider the same total dose of 60 Gy.

**Fig. 9:**
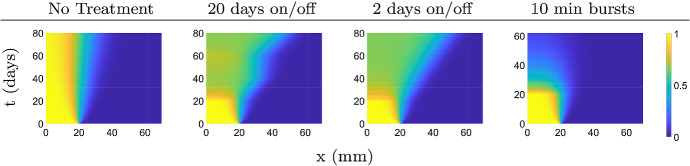
Top view of the solution surfaces of system (17) with $\delta = 0.3$ under fractionated treatments. Treatment begins on day 20 and the treatment type for each simulation is indicated by the column headings. The treatment simulations end when the total dose of 60 Gy is reached (Color figure online)

The simulations in Fig. 9 show that the invasion speed increases while on therapy, and returns to normal during treatment holidays. The invasion paradox is most prominent for 20/20 and 2/2 protocols, while the effect of the short bursts during fractionated treatment is limited because the time interval of increased cell death is too short to make a difference to the overall invasion of the tumor (Fig. 9).

### Treatment Dynamics on the Slow Manifold

**Fig. 10:**
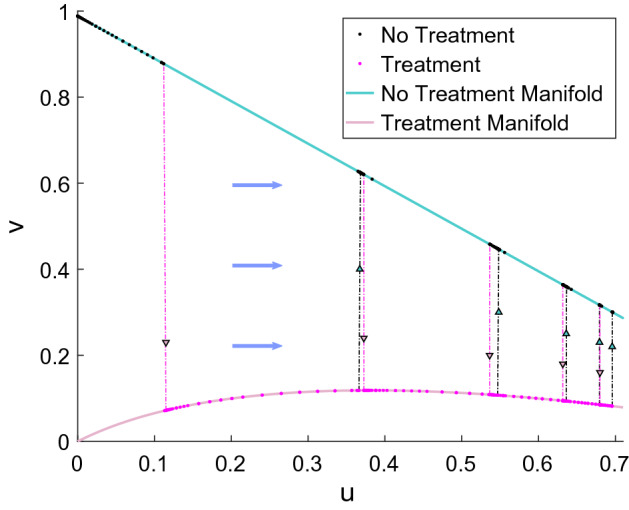
An example of solution movement along treatment and no-treatment manifolds from (17) with $\delta = 0.1$. The plot shows the densities *u*(*x*, *t*) (CSC) and *v*(*x*, *t*) (TC) at a fixed spatial location $x=21.97$ as functions of time. Here we consider a 2/2 treatment of 8Gy $\hbox {day}^{-1}$ for 17.5 treatment days starting on day 2. The blue arrows indicate the general direction of movement of the solutions (Color figure online)

It is interesting to consider the treatment dynamics in the context of the slow manifold analysis that we used in our theory. Active treatment changes the slow manifold because *M* in (6) depends on the death rate $\alpha$. Hence, upon the activation of treatment, we jump from the no-treatment manifold onto the treatment manifold, returning to the no-treatment manifold once treatment stops. We show an example of 2 days on and 2 days off treatment in Fig. 10. The blue curve is the no-treatment manifold and the light pink curve is the treatment manifold. In pink and black we see the jumps between manifolds when treatment is turned on (pink) or turned off (black). We notice the different speeds of growth on the corresponding manifolds, as the black parts on the no-treatment manifold are short, while the pink parts on the treatment manifold are wider, each representing a span of 2 days. In this case treatment is not able to control the tumor and we see a move of the dynamics to the right, that is, to a full cancer equilibrium.

## Discussion

Tumors are understood to be complex adaptive dynamic systems. Phenotype heterogeneity, with cancer stem cells on top of a differentiation hierarchy that receives feedback and competition from more mature cell types, further complicates this nonlinear system. We and others have previously shown that elevated levels of cell death may counterintuitively accelerate tumor growth, dubbed the tumor growth paradox (Enderling et al. 2009; Hillen et al. 2013; Brady-Nicholls et al. 2020). Herein, we investigated an additional angle of these dynamics and present simulation results of an agent-based and a differential equation modeling approach to elucidate the tumor invasion paradox. We have demonstrated that the model proposed by Hillen et al. (2013) reduces to a Fisher or Kolmogorov–Petrovsky–Piskunov-type equation on the slow manifold, which admits travelling wave solutions. By analyzing the travelling wave speeds, we showed that there exists an invasion paradox, where increasing the death rate of TCs increases the invasion speed of the tumor. Further, our analysis determined the specific conditions and cell death rates for when the invasion paradox occurs.

Of importance, we restricted the herein analyses on a generic tumor with a generic parameter set. While some of the parameters may have references in the literature to different tumor types, we focus on conditions when an invasion paradox occurs, without references to specific cancer types or treatment approaches. The comparison of continuous, intermittent, and fractionated therapies, however, may have strong support in prostate and brain cancers, as well as hormone therapy and radiation treatments (Zhang et al. 2017; Brady-Nicholls et al. 2020, 2021; Brueningk et al. 2021). We note that in our numerical investigations we focus on showing the cancer stem cells. However, there is still a TC compartment that needs to be included to infer the full tumor size. Furthermore, our analysis assumes a certain scaling of proliferation and radiation terms, and the full model needs to be studied if those scalings are not satisfied.

Increasing evidence shows de-differentiation in cancer populations under treatment (Hanahan 2022). For example in Lee et al. (2016) the production of treatment induced hypoxia inducible factors (HIF1$\alpha$ and HIF$2\alpha$) play a role in de-differentiating non-stem glioma cells to express stem cell markers. In (Dahan et al. 2014; Rhodes and Hillen 2016) a connection of survivin expression and de-differentiation in glioma was made. While we did not include de-differentiation in our model here, it is easy to argue that de-differentiation would further increase the effect of the invasion paradox.

Radiation and chemotherapies have many more effects than considered here. They destroy healthy tissue, stimulate an immune response, and modify the tumor microenvironment. All these factors will contribute to the tumor invasion. For example in Maggiorella et al. (2005) B16 melanoma cells were analyzed in an invasion assay and exposed to three levels of radiation (0 Gy, 4 Gy, and 8 Gy). In their invasion assay experiments increased radiation leads to significantly increased invasion speeds. In Kargiotis et al. (2008) they state in their abstract that “ *Recently, ionizing radiation has been shown to enhance invasiveness of surviving tumor cells ...*”. In that paper, the authors focus on the role of the uPA/uPAR (urokinase plasminogen activator and urokinase plasminogen activator receptor) pathway and its role in the increased invasion. In Jy (2018) a direct connection to cancer stem cells is made, as they say in their abstract that “*Transwell and micro array assays demonstrated that radioresistant GBM cells (GBM-R2I2) exhibit increased invasion and self-renewal abilities compared with parental GBM cells.*”

The analysis performed here leads to interesting mathematical questions as well. We formally apply geometric singular perturbation theory to our PDE model (4). In case of ordinary differential equations, such a theory was established through the Fenichel theorems (Hek 2010). These theorems state that if the slow manifold is normally hyperbolic and compact, then the full system has an invariant manifold that is close and diffeomorphic to the slow manifold. To understand the dynamics of the system it is sufficient to study the dynamics on the slow manifold, hence reducing the complexity. A corresponding theory has not yet been established for the case of PDEs. However, there are recent promising results in this direction, see (Bates et al. 2008; Kuehn and Soresina 2020; Avery and Scheel 2022).

Our continuum and ABM models are simplifications of cancer reality. Inside a human body, a cancer is exposed to the immune response, the tumor microenvironment, blood supply, chemokines and mechanical forces. These aspects are not included in our model, as our aim was to focus on the interplay of cancer stem cells and spatial spread. This is also the reason why we do not attempt to fit the model to a certain tumor type.

During our study, we performed many more simulations than shown. Looking at all these together we noticed a dichotomy in the model outcome, which can already be seen in Fig. 7c. That is, increased treatment kills more cancer cells and also extends the spatial cancer region. The question arises, which effect is more relevant. If the cancer is killed before any spatial effect is seen, then we are fine. However, if the cancer dies slowly while expanding in space, we might have created a worse situation. To further investigate this dichotomy we will have to calibrate and validate the model on specific medical data. This is a plan for future research.
